# The effects of quercetin on SW480 human colon carcinoma cells: a proteomic study

**DOI:** 10.1186/1475-2891-4-11

**Published:** 2005-03-04

**Authors:** Michael F Mouat, Kumar Kolli, Ronald Orlando, James L Hargrove, Arthur Grider

**Affiliations:** 1 Department of Foods and Nutrition, University of Georgia, Athens, Georgia 30602, USA; 2 Complex Carbohydrate Research Center, University of Georgia, Athens, Georgia 30602, USA

## Abstract

**Background:**

High fruit and vegetable intake is known to reduce the risk of colon cancer. To improve understanding of this phenomenon the action of different phytochemicals on colon cells has been examined. One such compound is quercetin that belongs to the group known as flavonoids. The purpose of this study was to determine the influence of quercetin on the proteome of the SW480 human colon adenocarcinoma cell line, specifically to identify proteins that could be the molecular targets of quercetin in its amelioration of the progression of colon cancer. To this end, two-dimensional gel electrophoresis and mass spectrometry were used to identify proteins that underwent a change in expression following treatment of the cells with 20 μM quercetin. This could elucidate how quercetin may reduce the progression of colon cancer.

**Results:**

Quercetin treatment of the SW480 human colon cancer cells was found to result in the decreased expression of three proteins and the increased expression of one protein. The identified proteins with decreased expression were type II cytoskeletal 8 keratin and NADH dehydrogenase Fe-S protein 3. The other protein with decreased expression was not identified. The protein with increased expression belonged to the annexin family.

**Conclusion:**

Several proteins were determined to have altered expression following treatment with quercetin. Such changes in the levels of these particular proteins could underlie the chemo-protective action of quercetin towards colon cancer.

## Background

Colorectal cancer is the third most common cancer diagnosed both in men and in women in the United States [1]. The American Cancer Society estimates that 104,950 new cases of colon cancer (48,290 men and 56,660 women) and 40,340 new cases of rectal cancer (23,530 men and 16,810 women) will be diagnosed in 2005 [1]. It is estimated that in the United States colorectal cancer will cause about 10% of all cancer-related deaths during 2005 with 56,290 deaths (28,540 men and 27,750 women) [1]. For 1998–2000 the lifetime probability of men in the United States developing cancer of the colon and rectum was 1 in 17; for women it was 1 in 18 [2].

Genetic factors account for only 10% of colorectal cancers [3,4] and thus environmental factors must also be involved in colon cancer development. Up to 80% of all colorectal cancer cases and deaths are attributable to diet [5] and such cases of colorectal cancer and related deaths may be prevented by dietary modifications [6]. Studies have suggested that a diet with an increased intake of fruit and vegetables correlates with a reduced risk of colorectal cancer [7,8]. For this, the chemical components of plants known as phytochemicals may be crucially involved [9]. Particular phytochemicals characterized by their phenolic ring structures are termed polyphenols and the most abundant and widely distributed of these are the flavonoids. Quercetin is the most widely distributed flavonoid found in foods, and is most abundant in apples, onions, black tea and red wine [10]. Studies *in vitro *and *in vivo *have suggested that quercetin may have a protective role against breast [11], lung [12], liver [13], ovarian [14] and colon [15-17] cancers.

Knowledge of how quercetin protects against cancer in general and colorectal cancer in particular could be gained by examining how quercetin affects the proteome of colon cancer cells. Specifically, the influence of quercetin on protein expression in the SW480 colon adenocarcinoma cell line could be instructive in elucidating the mechanisms underlying the protective role of the flavonoid against colon cancer. This would strengthen the scientific evidence for advocating a diet rich in fruit and vegetables to decrease the risk of developing colon cancer. Moreover, an identification of those proteins affected by quercetin could enhance understanding of the roles of these proteins in colon neoplasia. Use of proteomic techniques was therefore adopted in this study to determine the influence of quercetin treatment on protein expression in SW480 colon cancer cells. Quercetin treatment was found to result consistently in the decreased expression of three proteins and the increased expression of one protein. All, except one of the down-expressed proteins, were identified by mass spectrometry. Thus protein targets that could be the molecular basis of inhibition of colon cancer by quercetin were obtained.

## Methods

### Cell culture

SW480 human colon carcinoma cells (ATCC, Rockville, MD) were grown in Leibovitz's L-15 medium with 2 mM L-glutamine (ATCC, Rockville, MD), supplemented with 10% fetal bovine serum (Equitech-Bio Inc., Kerrville, TX). Cells were maintained in 75 cm^2 ^canted-neck flasks in 15 ml medium and incubated at 37°C without CO_2_. They were subcultured once per week at a ratio of 1:4.

### Quercetin treatment

When cells were about 90% confluent at seven days after passage the medium was discarded and replaced with medium containing 20 μM quercetin (Sigma, St. Louis MO). Cells were incubated for 48 h in the quercetin-containing medium at 37°C in air.

### Isolation of protein

Following incubation of the cells in quercetin for 48 h the medium was discarded and the cells washed three times for 1 min each time with phosphate-buffered saline. After complete removal of the final wash buffer, to each culture flask was added 240 μl boiling sample buffer I (0.3% sodium dodecyl sulfate (SDS), 200 mM dithiothreitol (DTT), 50 mM Tris-HCl, pH 8.0). The lysed cells were scraped together using a cell scraper (Fisher, Pittsburgh PA). The lysate from each culture flask was transferred to a 1.5 ml microfuge tube and heated for 5 min at 100°C. After chilling the tube on ice 24 μl (1/10 volume) of sample buffer II (50 mM magnesium chloride, 0.1% DNAse I, 0.025% RNAse A, 0.5 M Tris-HCl, pH 8.0) was added. Acetone was added to 80% (v/v) and the tube incubated on ice for 20 min. After centrifugation at 12,000 rpm for 10 min the supernatant was discarded and the pellet resuspended in 240 μl freshly prepared immobilized pH gradient (IPG) sample buffer (7 M urea, 2 M thiourea, 4% CHAPS, 1% DTT and 2% pH 3–10 ampholytes). The protein concentrations of the suspensions were measured using the method of Bradford with Bio-Rad reagent and bovine plasma gamma globulin as standard (Bio-Rad, Hercules, CA).

### Immobilized pH gradient electrophoresis

As the first dimension of two-dimensional electrophoresis Bio-Rad pH 3–10 immobilized pH gradient (IPG) strips were used to separate the cell proteins according to their isoelectric points. A volume of the cell protein solution containing 2 mg protein was mixed with rehydration buffer (6 M urea, 2 M thiourea, 2% CHAPS, 0.4% DTT and 0.5% pH 3–10 ampholytes) for a final volume of 300 μl per IPG gel. The IPG strips were loaded with the protein sample and the strips passively rehydrated for 16 h. Isoelectric focusing of the strips was at 250 V for 15 min followed by rapid ramping to10,000 V for 3 h and maintenance at the peak voltage for another 60,000 Vh. Current was limited to 50 μA per gel.

### Second dimension slab gel

The IPG strips were equilibrated with 6 M urea, 30% glycerol, 2% SDS, 1.55 M Tris, 2 mM tributylphosphine (TBP) and 0.00125% bromophenol blue (BPB) for 25 min with shaking at room temperature. The strips were rinsed in cathode buffer and applied to the top of 12% acrylamide slab gels. The cathode buffer contained 50 mM Tris, 384 mM glycine and 6.9 mM SDS. Low-melting point agarose (1% in cathode buffer) was layered over the strips. The anode buffer contained 25 mM Tris, 192 mM glycine and 3.5 mM SDS. The buffers were chilled to 4°C prior to use. A cooling plate maintained the system (Genomic Solutions, Ann Arbor, MI) at low temperature. The gels (22 cm × 22 cm × 1 mm) were run at 20 W per gel until the BPB dye front had migrated to within 1 cm from the bottom of the gel. The gels were stained with Coomassie blue.

### Quantification of protein spots

Second dimension gels were analyzed by Phoretix software (Nonlinear Dynamics, Newcastle, UK). Comparison between gels was achieved using normalized spot volumes. To begin with, differences greater or equal to 1.5-fold were considered to be significant. If these differences were subsequently reproduced in additional experiments, despite possibly being of lower magnitude, they were held to be consistent.

### Mass spectrometry

Differentially expressed protein spots were excised from the gels and digested with trypsin as reported previously [18]. Each gel plug was incubated with 150 μl of 50 mM ammonium bicarbonate containing 12.2 M acetonitrile. The gels were then dried and rehydrated with 5 μl of 50 mM ammonium bicarbonate containing 0.5 μg trypsin. This solution was added in 5 μl aliquots until the gel piece regained its original size. The gel was then covered with 50 mM ammonium bicarbonate and incubated overnight at 30°C. After the addition of 1.5 μl of 880 mM trifluoroacetic acid the peptides were extracted with 50 mM ammonium bicarbonate containing 14.6 M acetonitrile. The supernatants were dried to approximately 10 μl. The samples were each mixed with MALDI matrix solution prepared as 5 mg/ml α-cyano-4-hydroxycinnamic acid in a solution containing 1:1:1 acetonitrile, ethanol and 0.1% trifluoroacetic acid (pH 2.0). Each mixture was spotted on a MALDI target and allowed to air-dry. Peptide masses were then obtained by MALDI-TOF MS (Applied Biosystems 4700 Proteomics Analyzer). Calibration was performed with the same procedure using mixtures of peptides with known molecular masses.

MS/MS ions produced by MALDI-TOF from the tryptic peptides and the results of a Mascot search  were used to determine protein identification. The NCBInr database, i.e. the non-identical nr protein database of the National Center for Biotechnology Information, was utilized for the search.

## Results

### Two-dimensional gel electrophoresis

**Figure 1:**
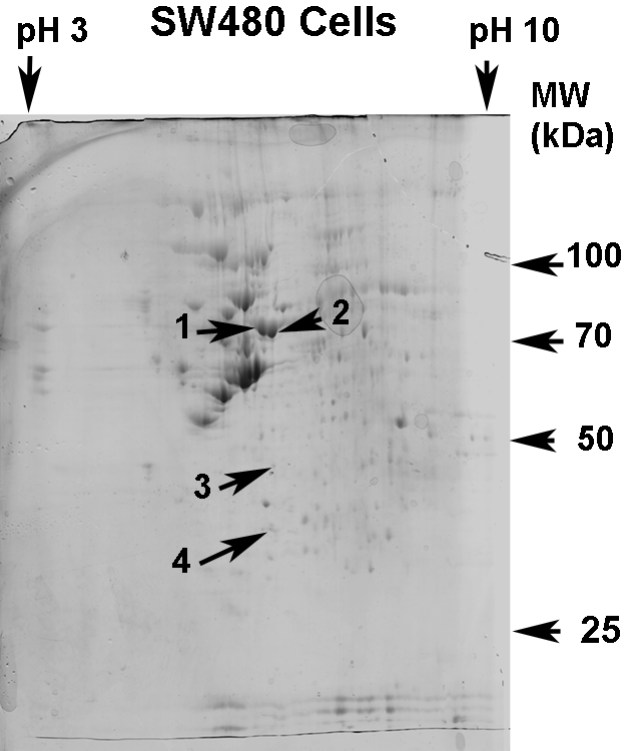
**Two-dimensional gel of protein from SW480 colon adenocarcinoma cells. **SW480 cells were cultured as described in **Methods**. In this representative case the cells were not treated with quercetin and were thus a control. Cells were harvested; protein was extracted and subjected to two-dimensional electrophoresis as described in **Methods**. The gel was stained with Coomassie blue. The protein spots indicated were those subsequently determined to be significant in the effects of quercetin.

**Figure 2:**
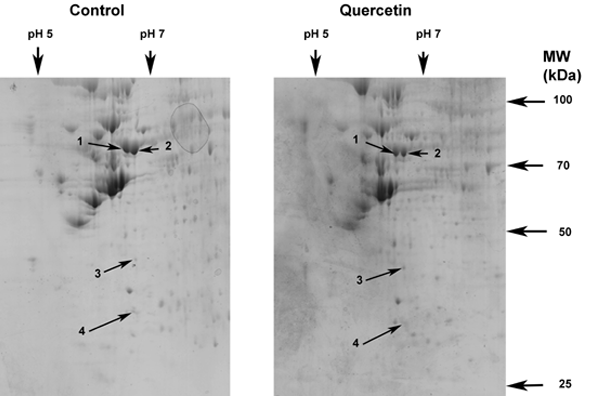
**Two-dimensional gels of protein from control and quercetin-treated cells. **After 7 days in culture SW480 cells were incubated for 48 h in medium containing A. 0.2% dimethylsulfoxide (DMSO), since DMSO was the vehicle in which stock quercetin had been dissolved, and B. 20 μM quercetin and 0.2% dimethylsulfoxide (DMSO). Cells were harvested, protein extracted and subjected to two-dimensional electrophoresis as described in **Methods. **The gels represent protein obtained from the control and quercetin-treated cells of one of four separate treatment groups. Treatment was identical for each group. The indicated protein spots differed in levels consistently for each of these treatment groups.

Fig. 1 demonstrates a gel obtained from two-dimensional gel electrophoresis of protein extracted from SW480 cells. More than 500 protein spots could be observed. The gels obtained from two-dimensional gel electrophoresis of quercetin-treated and untreated (control) SW480 cell proteins were compared (Fig. 2). Two mg protein was loaded in each case. Four proteins were revealed to have undergone a consistent change in expression following treatment with 20 μM quercetin for 48 h. Three proteins had a decreased and one protein an increased expression. The changes in these particular proteins were consistent between four separate experiments, i.e. four separate quercetin treatments coupled in each case with an untreated control. Two experiments were run concurrently and then another two on a different occasion. The proteins with altered expression following quercetin treatment, consistent between different experiments, are labeled 1–4 in Fig. 2. With observation solely by eye some of the differences may not be readily apparent, but they were verified by analysis of the gels using Phoretix 2-D analysis software (Table 1). The results in Table 1 are the means from analysis of the gels from two separate experiments conducted concurrently. In parentheses are the means from a second set of two experiments run concurrently at a different time to the first two experiments. The variation between the two sets of results can be attributed to the cells having been subcultured several times between the first and second sets of experiments. Furthermore, the cells could have been in a stage of growth for the second set of experiments different from that for the first. Despite these sources of variation, a consistent effect of quercetin in all four experiments was searched for. From these results proteins 1, 2 and 4 each had decreased expression following quercetin treatment of the cells. Protein 3 had increased expression.

**Table 1:** Effect of quercetin treatment of SW480 cells on the volume of protein spots on 2-D gels. The 2-D gels of protein from the untreated (control) and quercetin-treated SW480 cells were analyzed with Phoretix software. Normalized spot volumes were computed, averaged for two separate experiments run concurrently, and compared between the control and quercetin-treated gels. The 2-D gels obtained from one of the experiments are presented in Fig. 2. Results are the means, from the two experiments, for protein spots that differed in volume consistently in these two experiments and a subsequent two experiments. Alongside these results in parentheses are the mean results from the second set of two experiments.

**Protein spot number**	**Control volume**	**Quercetin volume**	**Increase/Decrease**	**Fold change**
1	6.61 (1.65)	2.89 (1.35)	Decrease	-2.29 (-1.22)
2	5.91 (4.18)	2.06 (3.26)	Decrease	-2.87 (-1.28)
3	0.118 (0.036)	0.243 (0.108)	Increase	+2.06 (+3.00)
4	0.411 (0.102)	0.088 (nd)	Decrease	-4.67 (-high)

### Mass spectrometry

The protein spots that were differentially expressed following quercetin treatment were excised from the gels and digested with trypsin. The tryptic digests were subjected to matrix-assisted laser desorption and ionization-time of flight (MALDI-TOF) mass spectrometry. The spectra were analyzed and the data entered into appropriate databases to identify the proteins. Table 2 lists the protein identities together with their functions. Protein number 1 could not be identified. Due to its proximity on the 2-D gels to protein number 2, i.e. type II cytoskeletal 8 keratin, it is possible that it is the same protein, but differing in its modification. It is known that this keratin is phosphorylated on serine residues; a process which is enhanced during epidermal growth factor stimulation and mitosis.

**Table 2:** Proteins differentially expressed following quercetin treatment of SW480 cells. Protein spots determined to be present at different levels on gels following quercetin treatment of the SW480 cells were excised from the gels, digested with trypsin and subjected to MALDI-TOF mass spectrometry as described in **Methods**. From the mass spectra the identities of the proteins were determined using the appropriate databases.

**Number from gel**	**Protein**	**Function**
2	Keratin, type II cytoskeletal 8	Cytoskeletal structure, angiogenesis-related
3	Annexin family	Ca^2+ ^and phospholipid binding, regulation of exocytic and endocytic pathways
4	NADH dehydrogenase (ubiquinone) Fe-S protein 3	Transfer of electrons from NADH to the respiratory chain

## Discussion

Four proteins were found to have their expression consistently altered on quercetin treatment of SW480 colon carcinoma cells. Three of these proteins were down-regulated and one was up regulated following exposure to quercetin. Using mass spectrometry three of the proteins were identified. Those that were down-regulated were type II cytoskeletal 8 keratin and NADH dehydrogenase (ubiquinone) Fe-S protein 3. The up-regulated protein belonged to the annexin family.

Type II cytoskeletal 8 keratin (keratin 8) in another proteomic study has been found to have a significantly lower abundance in the normal mucosa compared with the adjacent colon tumor for one specific patient for two out of three gel protein features identified as keratin 8 [19]. For the same patient, keratins 18 and 19 also had significantly lower concentrations in the normal mucosa compared with the adjacent tumor. Similarly, with paired samples of normal colon mucosa and adenocarcinomas derived from 27 patients, proteomic analysis revealed that keratin 18 was at a significantly lower level in the normal mucosa compared with the tumor tissue [20]. In these respects down regulation of keratin 8 by quercetin treatment of SW480 cells would be akin to the cells becoming more "normal", i.e. less tumorigenic. Further support for this contention derives from analysis using the cDNA macroarray technique of the differential gene expression in isolated human colorectal cancer and respective normal mucosa from two patients [21]. The tumors showed up-regulation of expression of type II cytoskeletal 8 keratin and other angiogenesis-related genes to over 5-fold the levels in normal mucosa. In the present study the down-regulation of type II cytoskeletal 8 keratin by quercetin may therefore demonstrate the decreased potential for angiogenesis and hence reduction of any tumor.

Further support for the reduction in expression of type II cytoskeletal 8 keratin following quercetin treatment of SW480 colon cancer cells being reflective of decreased neoplasia is derived from a proteomic analysis of lung adenocarcinomas [22]. Cytoskeletal 8 keratin, in addition to other keratins, had reduced expression in normal lung samples compared with lung adenocarcinomas. Furthermore, several isoforms of cytoskeletal 8 keratin were identified. These had differing pI, but similar molecular weights, and apparently result from differing extents of posttranslational phosphorylation [22]. This supports our contention that unidentified protein 1 in the present study may be an isoform of type II cytoskeletal 8 keratin. A crucial role for this keratin in the malignant phenotype is suggested by studies on its increased expression by transfecting stratified epithelial cells with the cytoskeletal 8 keratin gene [23] and the epidermal consequences for transgenic mice expressing the human keratin 8 [24]. In each case neoplastic transformation of the cells to the malignant phenotype occurred.

NADH dehydrogenase (ubiquinone) Fe-S protein 3 is a component of complex 1 of the mitochondrial electron transport chain. It is involved in transfer of electrons from NADH to ubiquinone in the respiratory chain. A similar component of complex 1 of the respiratory chain, NADH-ubiquinone oxidoreductase, has been determined from a proteomic analysis to be present in colon tumor tissue and the surrounding normal mucosa [19]. Interestingly, its abundance was higher in the tumor than in the normal mucosa. This suggests that the lower level of NADH dehydrogenase (ubiquinone) Fe-S protein 3 in our quercetin-treated colon cancer cells compared with its level in the untreated cells is linked to a decreased tumorigenicity of the cells following quercetin treatment. Furthermore, NADH dehydrogenase (ubiquinone) has also been determined by others to have a decreased abundance in HT-29 human colon cancer cells following exposure for 24 h to 150 μM quercetin [25]. This supports what we determined for NADH dehydrogenase (ubiquinone) Fe-S protein 3. The implication is that the consequent reduction in oxidative phosphorylation of substrates by quercetin is associated with decreased tumorigenicity.

Following quercetin treatment, the only protein to be up-regulated belonged to the annexin family. In another proteomic study annexins I, III, IV and V have been determined to have a greater abundance in normal mucosa compared with neighboring tumor tissue [19]. Thus the rendering of the SW480 colon cancer cells less tumorigenic by treatment with quercetin could be reflected by the increased abundance of members of the annexin family. To strengthen this argument the level of annexin II, a protein that inhibits cell migration, was found to increase in HT-29 human colon cancer cells following treatment with quercetin for 24 h and 48 h [25]. Inhibiting the ability of the cells to migrate would contribute to explaining the anti-cancer activity of quercetin. In addition, annexin I, which promotes apoptotic cell engulfment, increased in HT-29 cells exposed to quercetin for 48 h. A role for apoptosis was also suggested when annexin IV, a key regulator of apoptosis, was found to increase in NCOL-1 human preneoplastic colonocytes following treatment with quercetin for 24 h [26]. Apoptosis, mediated by members of the annexin family, may therefore also underlie the anti-cancer activity of quercetin.

## Conclusion

Three proteins have been identified as potential molecular targets for the proposed action against colon cancer of quercetin the plant flavonoid. The application of proteomic techniques demonstrated the response of these proteins to quercetin treatment of colon cancer cells. Other research in the field supported the response of each of these proteins to quercetin. This serves to validate the previous work. Furthermore, the responses could be rationally described in terms of an anti-cancer action. This strengthens earlier findings of quercetin's protection against colon cancer using cell culture and animal models. A basis is thus provided for further research with quercetin and perhaps even advancement to studies with humans. An intriguing aspect to consider is whether quercetin could form the basis of directed drug design to yield drugs with greater efficacy against the identified molecular targets and thereby provide an effective treatment of colon cancer.

## List of abbreviations used

NADH, nicotinamide adenine dinucleotide, reduced;

MALDI-TOF, matrix-assisted laser desorption and ionization-time of flight;

MS, mass spectrometry

2-D, two-dimensional;

SDS, sodium dodecyl sulfate;

DTT, dithiothreitol;

IPG, immobilized pH gradient;

CHAPS, 3-[(3-cholamidopropyl) dimethylammonio]-1-propane-sulfonate;

TBP, tributylphosphine;

BPB, bromophenol blue;

DMSO, dimethylsulfoxide;

NCBI, National Center for Biotechnology Information.

## Competing interests

The authors declare that they have no competing interests.

## Authors' contributions

MFM treated the cells, performed the two-dimensional electrophoresis and gel analysis, submitted protein spots for mass spectrometry, undertook the literature search on the identified proteins, drafted the manuscript, submitted the manuscript and completed all the necessary revisions to make it acceptable for publication. KK carried out trypsin digestion of the protein spots and undertook the mass spectrometry. RO provided oversight for the mass spectrometry. JLH and AG participated in conception and design of the study. All the authors read and approved the final manuscript.
